# Histologic heterogeneity in synchronous bilateral breast cancer: a case report and literature review

**DOI:** 10.3389/fonc.2026.1936986

**Published:** 2026-10-01

**Authors:** Xuedi Yuan, Wen Liu, Xiuxia Liu, Chuyun Sun, Yuwen Cao

**Affiliations:** 1 Department of Pathology, Shihezi University of Medicine, Shihezi, Xinjiang, China; 2 Department of Pathology, The First Affiliated Hospital of Shihezi University, Shihezi, Xinjiang, China; 3 Department of Ultrasound Medicine, The First Affiliated Hospital of Shihezi University, Shihezi, Xinjiang, China

**Keywords:** case report, discordant histologic subtypes, histologic heterogeneity, invasive ductal carcinoma, invasive lobular carcinoma, synchronous bilateral primary breast cancer

## Abstract

Synchronous bilateral primary breast cancer (sBPBC) accounts for 2.9%–3.9% of newly diagnosed breast cancers, while cases with discordant histologic subtypes are exceptionally rare. We report on a 59-year-old woman with synchronous left-sided invasive ductal carcinoma (IDC) and right-sided invasive lobular carcinoma (ILC). The patient underwent bilateral breast-conserving surgery with sentinel lymph node biopsy, followed by adjuvant radiotherapy and endocrine therapy. No recurrence or metastasis was observed during the 12-month follow-up. To place the present case in the context of the existing literature, we comparatively reviewed seven previously reported cases of synchronous bilateral IDC and ILC. The comparison illustrated considerable heterogeneity in the clinicopathological characteristics, molecular profiles, nodal involvement, treatment strategies, and clinical outcomes. These findings support independent characterization of each breast lesion and individualized treatment planning based on the features of both tumors.

## Introduction

Bilateral primary breast cancer (BPBC) is uncommon. Based on the interval between diagnoses, BPBC is classified as either synchronous bilateral primary breast cancer (sBPBC) or metachronous bilateral primary breast cancer (mBPBC). The World Health Organization uses a 3-month cutoff to distinguish synchronous from metachronous disease. sBPBC comprises an estimated 2.9%–3.9% of newly diagnosed breast cancers (1, 2). Cases of sBPBC with discordant histologic subtypes, including invasive ductal carcinoma (IDC) in one breast and invasive lobular carcinoma (ILC) in the contralateral breast, have previously been reported. However, the available literature remains limited to sporadic case reports, and the clinicopathological characteristics, molecular profiles, diagnostic features, treatment strategies, and outcomes of these cases have not been well characterized comparatively. Herein, we report on a case of sBPBC with left-sided IDC and right-sided ILC and review previously reported cases with similar histologically discordant presentations to summarize their clinicopathological and molecular heterogeneity, diagnostic challenges, variations in therapeutic strategies, and clinical outcomes.

## Case presentation

A 59-year-old postmenopausal woman presented to our institution with a palpable left breast mass incidentally discovered during routine self-examination. Physical examination demonstrated a firm, mobile, well-circumscribed mass measuring approximately 2.5 cm at the 10 o’clock position of the left breast. Palpation of the right breast revealed no grossly palpable lesions. Bilateral axillary and supraclavicular lymph node examination revealed no lymphadenopathy or tenderness. Notably, the patient reported a significant personal history of multiple uterine leiomyomas diagnosed in 2004. She denied any family history of breast cancer, ovarian cancer, or other hereditary malignancies.

Breast Doppler ultrasound revealed a 2.6-cm × 1.8-cm × 2.4-cm heterogeneous hypoechoic nodule at the 10 o’clock position of the left breast, categorized as Breast Imaging Reporting and Data System (BI-RADS) 4C (**Figure 1A**). Small hypoechoic lesions in the right breast showed relatively benign-appearing features and were categorized as BI-RADS 3. Given the highly suspicious lesion in the left breast, further bilateral imaging assessment was performed. Mammography subsequently identified an irregular, spiculated mass with punctate calcifications in the left breast, categorized as BI-RADS 4C (**Figures 2A, B**), and a focal asymmetric density in the right breast, categorized as BI-RADS 4A (**Figures 2C, D**). Repeat ultrasonography performed later the same day identified a 1.0-cm × 0.9-cm suspicious nodule at the 10 o’clock position of the right breast, categorized as BI-RADS 4C (**Figure 1B**). Ultrasound examination of the bilateral axillae revealed no evidence of pathologic lymphadenopathy. Contrast-enhanced MRI identified a patchy abnormal signal in the left breast, categorized as BI-RADS 5 (**Figure 3A**), and an irregular nodular signal categorized as BI-RADS 4C in the right breast (**Figure 3B**). Subsequently, the patient underwent ultrasound-guided core needle biopsies of both breast masses. Pathological evaluation confirmed IDC (**Figures 4A, B**) in the left breast and ILC (**Figures 4C, D**) in the right breast. Given her history and her own preference, the patient proceeded with bilateral breast-conserving surgery with sentinel lymph node biopsy.

**Figure 1:**
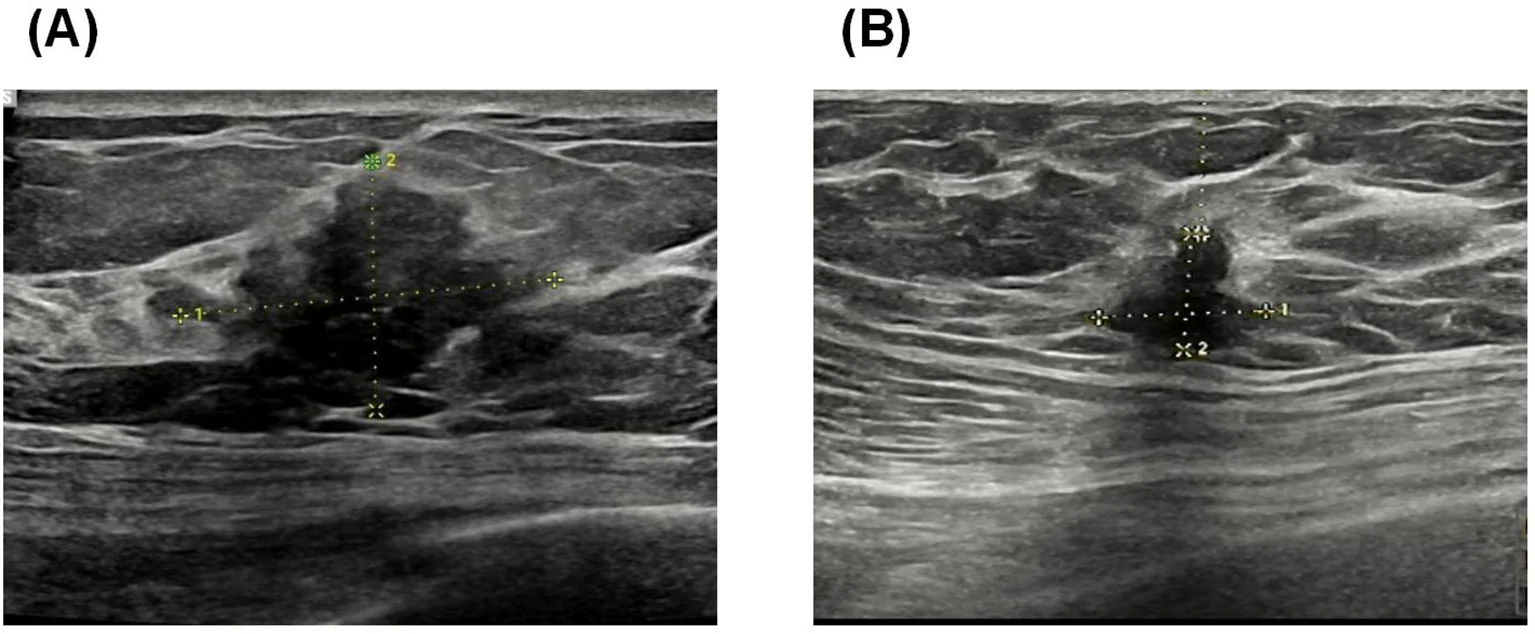
Breast Doppler ultrasound findings. **(A)** Left breast lesion located at the 10 o’clock position, measuring 2.6 cm × 1.8 cm × 2.4 cm and categorized as BI-RADS-US 4C. **(B)** Right breast lesion located at the 10 o’clock position, measuring 0.9 cm × 1.0 cm and categorized as BI-RADS-US 4C after re-evaluation.

**Figure 2:**
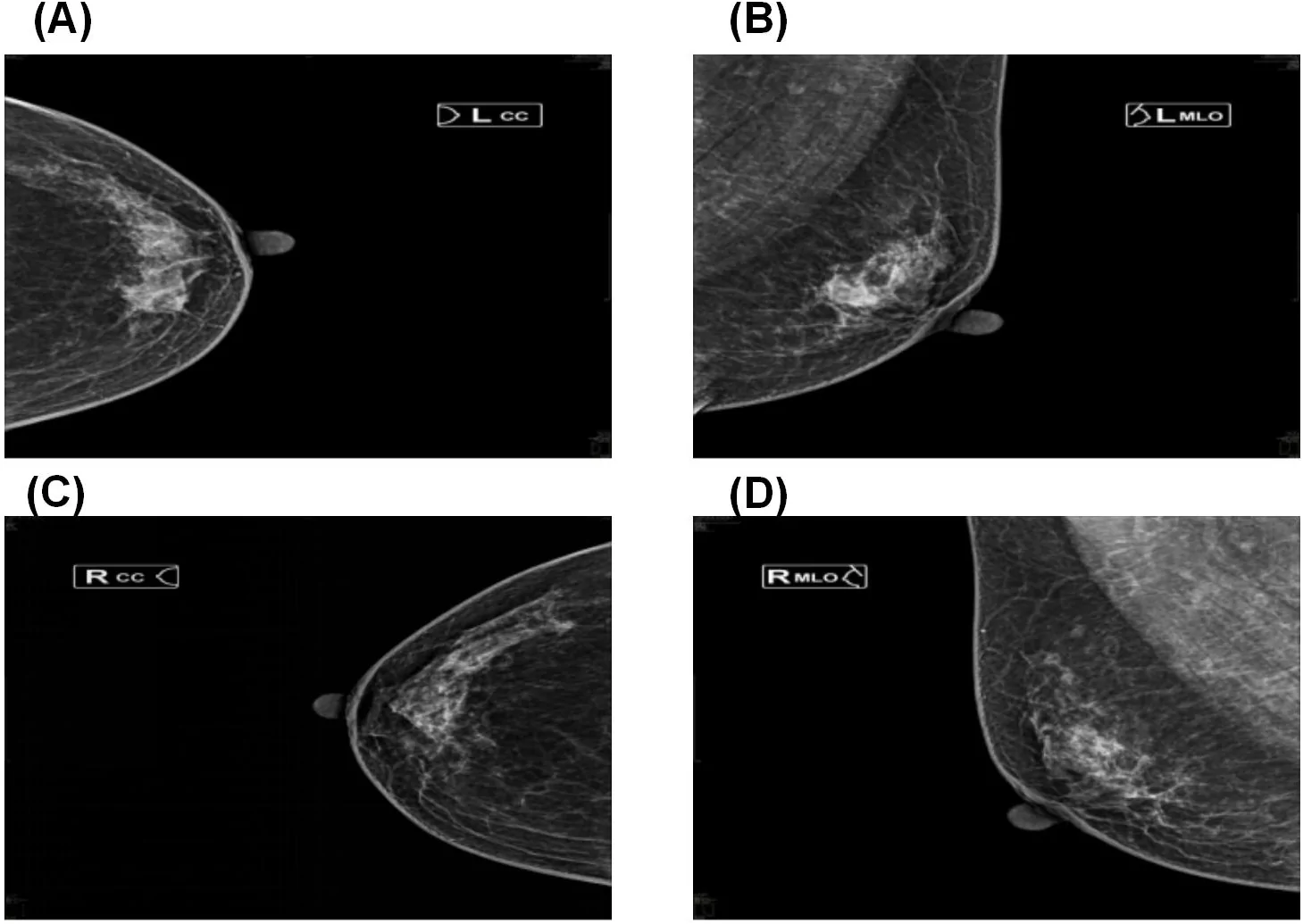
Mammographic findings of bilateral breast lesions. **(A, B)** Craniocaudal (*CC*) and mediolateral oblique (*MLO*) views of the left breast demonstrating an irregular spiculated mass with punctate calcifications. **(C, D)** Craniocaudal (*CC*) and mediolateral oblique (*MLO*) views of the right breast showing focal asymmetric density.

**Figure 3:**
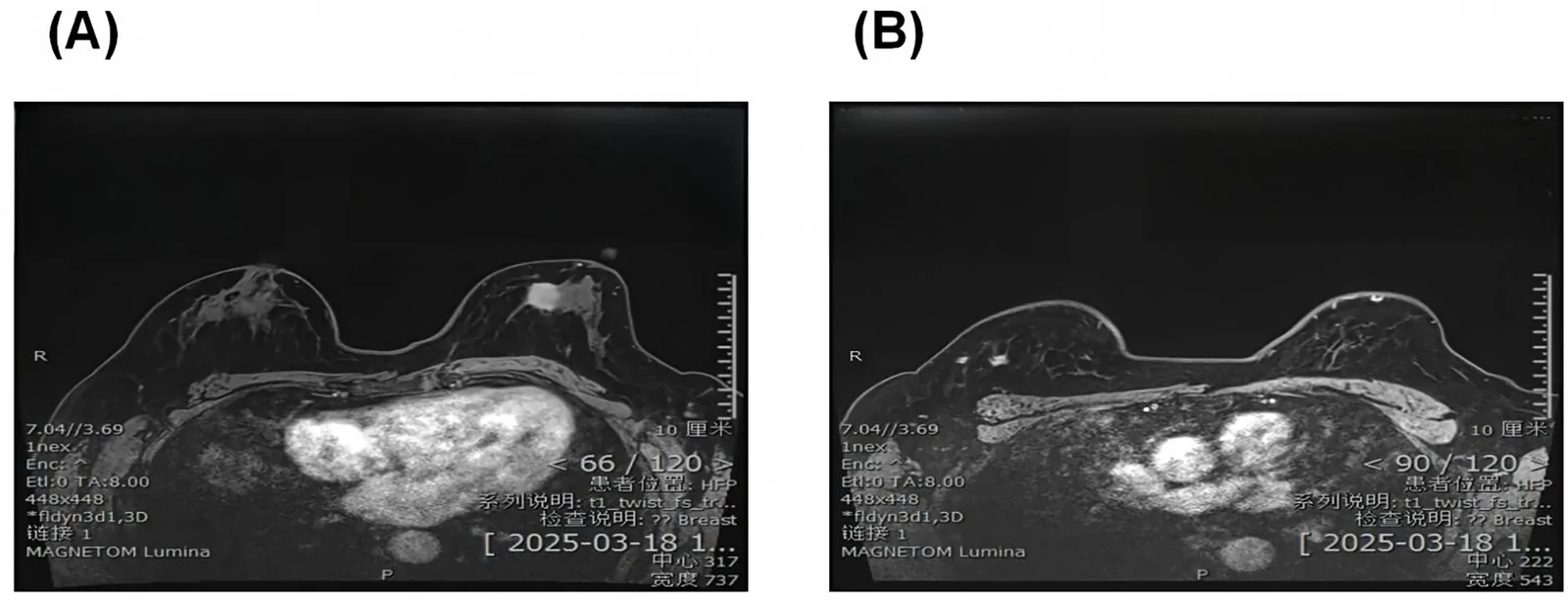
Breast magnetic resonance imaging findings. **(A)** Contrast-enhanced MRI of the left breast demonstrating a patchy abnormal enhancing lesion categorized as BI-RADS 5. **(B)** Contrast-enhanced MRI of the right breast demonstrating a nodular enhancing lesion categorized as BI-RADS 4C.

**Figure 4:**
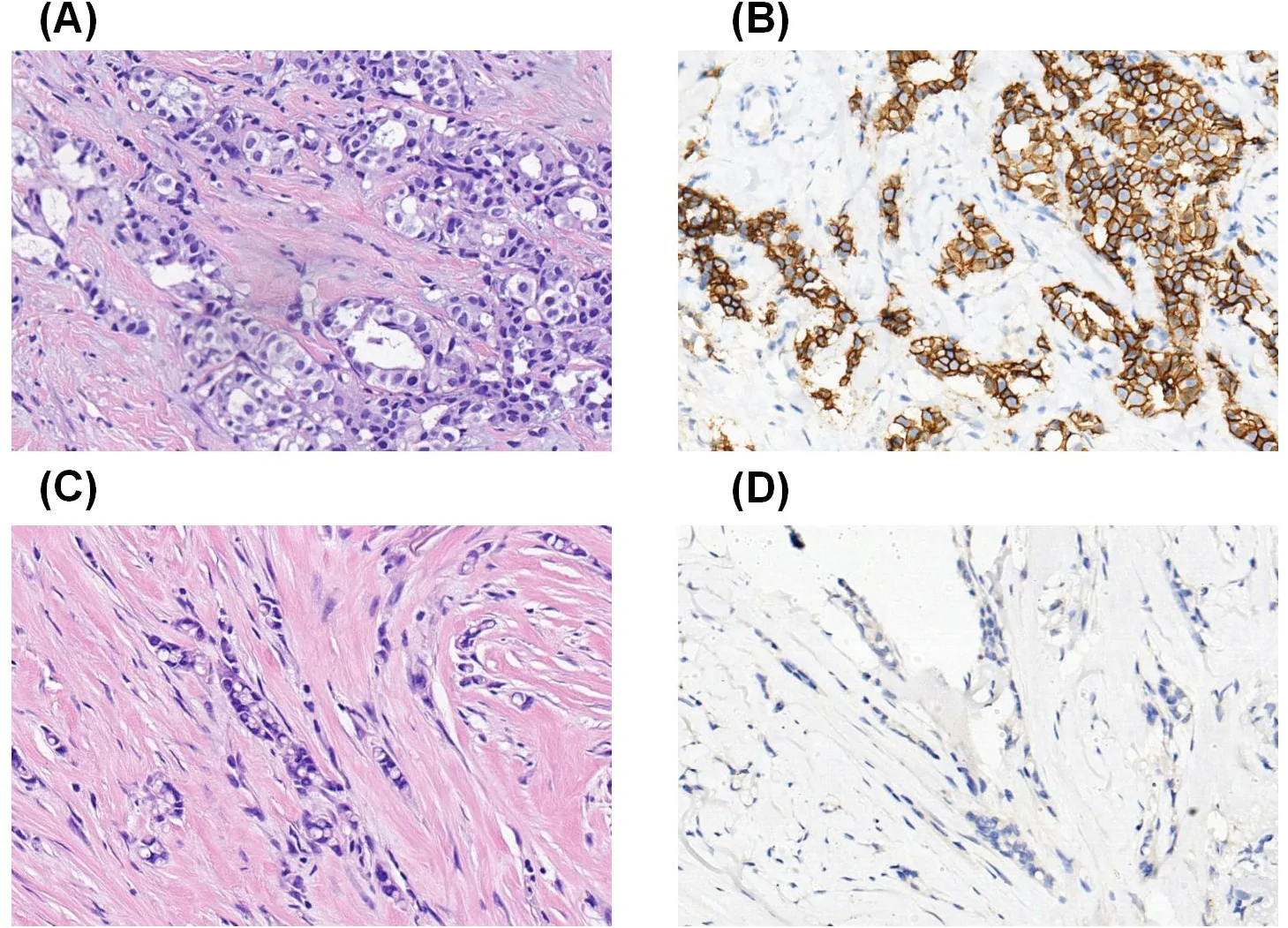
Histopathological and immunohistochemical findings of bilateral breast tumors. **(A)** Hematoxylin and eosin staining of the left breast tumor demonstrating invasive ductal carcinoma (×200). **(B)** Immunohistochemical staining showing positive E-cadherin expression in the left breast tumor (×200). **(C)** Hematoxylin and eosin staining of the right breast tumor demonstrating invasive lobular carcinoma (×200). **(D)** Immunohistochemical staining showing loss of E-cadherin expression in the right breast tumor (×200).

Postoperative pathological examination of the left breast revealed a 2.6-cm × 2.2-cm × 1.6-cm grade 1 IDC, with estrogen receptor (ER) positivity of 85%, progesterone receptor (PR) positivity of 85%, and a Ki-67 proliferation index of 10%. The right breast lesion was a 1.8-cm × 1.5-cm × 1.2-cm grade 2 ILC, with ER positivity of 70%, PR positivity of 30%, and a Ki-67 proliferation index of 8%. Bilateral masses were HER2-negative, and sentinel lymph nodes appeared normal and showed no metastatic carcinoma. Lymphovascular invasion was not identified in either specimen. All surgical margins were free of tumor, confirming complete resection. Preoperative chest and head computed tomography (CT) and abdominal ultrasonography revealed no evidence of distant metastasis. According to the eighth edition of the American Joint Committee on Cancer (AJCC) staging system, the left-sided IDC was classified as pT2N0M0, whereas the right-sided ILC was classified as pT1cN0M0.

The perioperative course was uneventful. The patient was evaluated by a multidisciplinary team, which comprehensively considered her dual diagnosis of IDC and ILC and her molecular profile. Based on the 2026 Guidelines for Breast Cancer Diagnosis and Treatment by the China Anti-Cancer Association (3) and international consensus guidelines, endocrine therapy with the aromatase inhibitor letrozole was initiated as an adjuvant treatment for this postmenopausal patient with bilateral hormone receptor-positive tumors. Genomic recurrence testing was not performed. Since both tumors were HER2-negative and showed negative sentinel lymph nodes, low histologic grades, and low Ki-67 proliferation indices, the patient was considered to have a relatively low clinicopathological risk of recurrence. After multidisciplinary discussion, adjuvant chemotherapy was not administered. Standard bilateral adjuvant radiotherapy was administered following breast-conserving surgery to reduce locoregional recurrence risk. Given the need for long-term endocrine therapy, letrozole was initiated at a dose of 2.5 mg orally once daily, with a planned treatment duration of 5 years. Letrozole was continued during and after radiotherapy. During the 12-month follow-up period, no dose reduction, treatment interruption, or change in endocrine therapy was required. The patient remained adherent to treatment and reported no clinically significant treatment-related adverse events. At the 12-month follow-up, physical examination and breast ultrasonography showed no evidence of locoregional recurrence, and no clinical evidence of distant metastasis was identified. The key events in the patient’s diagnostic evaluation, treatment, and follow-up are summarized in **Table 1**.

**Table 1:** Timeline of the clinical course.

Date	Clinical event
7 March 2025	The patient presented with a self-detected left breast mass. Ultrasonography identified a BI-RADS 4C lesion in the left breast.
17 March 2025	The patient was admitted for further evaluation. Mammography and repeat ultrasonography identified suspicious bilateral breast lesions, with the right breast lesion categorized as BI-RADS 4A on mammography and BI-RADS 4C on ultrasonography.
18 March 2025	Ultrasound-guided core needle biopsy of the left breast lesion was performed.
20 March 2025	MRI demonstrated a BI-RADS 5 lesion in the left breast and a BI-RADS 4C lesion in the right breast. Ultrasound-guided core needle biopsy of the right breast lesion was performed.
25 March 2025	Histopathological examination of the core needle biopsies confirmed IDC in the left breast and ILC in the right breast. Bilateral breast-conserving surgery was planned after clinical evaluation and discussion with the patient.
26 March 2025	Bilateral breast-conserving surgery with bilateral sentinel lymph node biopsy was performed. Intraoperative frozen sections showed negative surgical margins and no sentinel lymph node metastasis.
31 March 2025	The patient was clinically stable and discharged, with postoperative follow-up arranged.
28 April 2025	Adjuvant endocrine therapy with letrozole (2.5 mg orally once daily) was initiated.
July–October 2025	Bilateral adjuvant breast radiotherapy was administered (25 fractions). Letrozole was continued during radiotherapy.
12-month follow-up	The patient remained on letrozole without dose reduction, treatment interruption, or clinically significant treatment-related adverse events. No locoregional recurrence or distant metastasis was detected.

IDC, invasive ductal carcinoma; ILC, invasive lobular carcinoma.

### Patient perspective

“When I learned that I had cancers in both breasts with different histological types, I felt very worried and confused. When discussing the surgical options, I was informed that breast-conserving surgery was feasible for both tumors. As I was also concerned about preserving my body image, I discussed the options with my family and ultimately chose bilateral breast-conserving surgery. I was relieved when the postoperative pathology showed negative surgical margins and no lymph node metastasis. I am grateful for the care provided by my medical team and feel more confident about the future”.

### Literature search and case selection

To place the present case in the context of the existing literature, PubMed and the China National Knowledge Infrastructure (CNKI) were searched from database inception through to 31 December 2025. PubMed was searched using combinations of the following terms: (“bilateral primary breast cancer” OR “synchronous bilateral breast cancer”) AND (“discordant” OR “discordance”). Corresponding Chinese terms were used to search CNKI. The reference lists of relevant articles were also manually screened to identify additional eligible reports.

Reports were eligible for inclusion if they described sBPBC with histologically confirmed IDC in one breast and ILC in the contralateral breast and provided sufficient patient-level clinicopathological information for data extraction. Reports lacking sufficient patient-level clinicopathological information were excluded. No language restrictions were applied. The full texts of potentially eligible reports were assessed for eligibility. Data on patient characteristics, histological type, molecular subtype, lymph node status, treatment, and clinical outcomes were extracted from the included reports and summarized descriptively. Following screening, seven previously reported cases met the eligibility criteria and were included in a descriptive comparison with the present case (**Table 2**).

**Table 2:** Summary of the clinicopathological characteristics and treatment prognosis of synchronous bilateral primary breast cancer (sBPBC) cases with histological inconsistency.

Case	Age at diagnosis (years)	Personal medical history	Family history	Left breast	Right breast	Lymph node status	Treatment principles and strategies	Main findings and outcomes
				histological/molecular subtype	histological/molecular subtype			
Present case	59	Multiple uterine leiomyomas	Mother with breast fibroadenoma	IDC Luminal	ILC Luminal	Bilateral SLNs: negative	BCS+SLNB–RT+ET	No recurrence or metastasis
Rao et al. (4)	59	Uterine leiomyoma, right mammary adenosis	None	ILC Luminal	IDC HER2-positive	R axillary nodes: 15/15 positive; L axillary nodes: 0/19	BMRM(ALND)–CT+TT–RT–ET	Vertebral bone metastasis of ILC after 9 years
Al-Bitar et al. (5)	46	None	None	IDC Luminal	ILC Luminal	L axillary nodes positive; R axillary nodes 2/19 positive with ECE	NACT–LRM+ALND–RT+ET	Right ILC resistance to NACT, right breast surgery pending
Peshin et al. (6)	61	Borderline diabetes, hypertension, and CAD	Grandmother and aunt with breast cancer; father with colon cancer	ILC Luminal	IDC Luminal	R SLN: 1 positive; L SLN: negative	BM+SLNB–RT(R)–ET+CDK4/6 inhibitor	Genetic and molecular profiling analysis
Brock et al. (7)	47	Smoking history	None	IDC Luminal	ILC Luminal	L axillary nodes: positive, resolved after NACT; L SLN: positive	NACT–BM+L SLNB+reconstruction–CT+RT+ET	Receptor discordance between primary tumor and metastatic node
Copur et al. (8)	48	NR	Mother with breast cancer and colon cancer	ILC Luminal	IDC TNBC	R axillary nodes: positive, negative after NACT; L SLN: 1 positive	NACT–BMRM (R ALND+L SLNB, followed by ALND)–RT+ET	IDC achieved complete response to neoadjuvant therapy; ILC had residual disease
Benkerroum et al. (9)	48	NR	None	IDC HER2-positive	ILC HER2-positive	Bilateral axillary and extra-axillary lymphadenopathy	CT+TT	ILC with cervical metastasis as initial presentation, multiple metastases, died after 2 years
Wang (10)	61	NR	NR	IDC HER2-positive	ILC HER2-positive	Bilateral axillary nodes: negative	BMRM–RT+CT	NR

CAD, coronary artery disease; IDC, invasive ductal carcinoma; ILC, invasive lobular carcinoma; TNBC, triple-negative breast cancer; ECE, extracapsular extension; BCS, breast-conserving surgery; SLNB, sentinel lymph node biopsy; ALND, axillary lymph node dissection; BMRM, bilateral modified radical mastectomy; LRM, left radical mastectomy; BM, bilateral mastectomy; RT, radiotherapy; ET, endocrine therapy; CT, chemotherapy; TT, targeted therapy; NACT, neoadjuvant chemotherapy; R, right; L, left; NR, not reported.

## Discussion

### Clinical diagnostic challenges in this case

In this case, the right ILC lesion measuring 1.0 cm × 0.9 cm was not palpable on physical examination. The absence of a palpable mass may partly reflect both its small size and the characteristic infiltrative growth pattern of ILC, in which tumor cells typically infiltrate as single cells or linear cords with a relatively limited stromal reaction, potentially resulting in less conspicuous clinical and imaging findings. The initially low-suspicion appearance of the contralateral lesion further illustrates the diagnostic challenge posed by small, clinically occult ILC. Serial multimodal imaging progressively increased suspicion for malignancy and ultimately prompted tissue diagnosis. MRI has been reported to be valuable for further characterization and assessment of the disease extent in ILC (11, 12). When a highly suspicious lesion is identified in one breast, the contralateral breast should be carefully and independently evaluated, and initially low-suspicion findings should be reassessed when warranted using complementary imaging modalities.

### Analysis of the clinicopathological features

In this case series of eight sBPBC patients with discordant histology, the median age at diagnosis was 53 years (range, 46–61 years). Among the eight patients with bilateral breast cancer, the distribution of IDC and ILC showed no apparent laterality preference. Notably, despite histological discordance between bilateral tumors, molecular subtype concordance was observed in six of the eight cases: both tumors were of luminal type in four cases and were HER2-positive in two cases. When analyzed by histological type, the ILC tumors comprised six luminal-type and two HER2-positive tumors, whereas the IDC tumors comprised four luminal-type, three HER2-positive, and one triple-negative tumor. The predominance of the luminal subtype among ILC tumors is broadly consistent with the molecular characteristics of ILC reported in The Cancer Genome Atlas (TCGA)-based analyses (13). Regarding regional lymph node involvement, nodal metastasis was reported in six of the eight patients (75%). Based on the laterality of the involved regional lymph nodes, nodal involvement was documented in six of eight IDC tumors and in three of eight ILC tumors. Although regional nodal involvement was numerically more frequent in IDC than in ILC in this series, this observation should be interpreted with caution given the small sample size and the heterogeneity in nodal assessment across the reported cases. A recent large-scale study reported no significant difference in the axillary lymph node metastasis rates between ILC and IDC (14).

### Medical history and hereditary considerations

The present patient had a personal history of multiple uterine leiomyomas, a finding also documented in a previously reported case of histologically discordant sBPBC by Rao et al. (4). Given the hormone-responsive nature of uterine leiomyomas, this co-occurrence is of clinical interest. Whether it has any relevance to the hormonal background of sBPBC remains to be explored. In the present case, the patient denied any significant family history of breast cancer or other hereditary malignancies, and germline genetic testing was not performed. Across the previously reported cases, family history was heterogeneous and incompletely reported across the eight cases. Two previously reported patients had a notable family history of malignancy. In one case, in which the patient’s grandmother and aunt had breast cancer and her father had colon cancer (6), genetic testing, including *BRCA1*/*2* evaluation, was initiated, although the final result was not reported. In another case, the patient’s mother had a history of both breast and colon cancers, and genetic testing for *BRCA1* and *BRCA2* mutations was negative (8). More broadly, hereditary predisposition is an important consideration in the assessment of bilateral breast cancer. Established breast cancer susceptibility genes include *BRCA1*, *BRCA2*, *PALB2*, *CHEK2*, and *TP53* (15). The occurrence of breast and colorectal cancers within the same family may warrant consideration of hereditary cancer predisposition in an appropriate clinical context. However, family history alone is insufficient to identify a specific hereditary cancer syndrome. Genetic counseling and germline multigene testing should be based on established clinical criteria incorporating personal and family cancer history and other relevant clinical features (16).

### Clonal origin hypothesis

Regarding the clonal relationship between bilateral breast tumors, two principal possibilities have been proposed: independent primary tumors arising from distinct clones and monoclonal metastatic spread (17, 18). The clonal relationship between bilateral tumors could not be established in either the present case or the seven previously reported cases included in this review because comparative genomic profiling of paired bilateral tumors was unavailable. Molecular approaches, including X-chromosome inactivation analysis, *TP53* mutation analysis, allelic imbalance analysis, and whole-exome sequencing, have been used to investigate the clonal relationship between bilateral breast tumors (19, 20). Such comparative molecular analyses provide a more direct basis for assessing clonality, whereas clinicopathological differences primarily reflect biological heterogeneity between bilateral tumors. For example, in the case reported by Copur et al. (8), the triple-negative IDC component achieved a complete response to neoadjuvant chemotherapy, whereas residual disease persisted in the luminal ILC component. This discordant treatment response highlights biological differences between the two tumors, although determination of their clonal relationship would require direct molecular comparison.

### Individualized therapeutic strategies

The treatment of sBPBC requires an individualized approach. In the present case, the bilateral tumors were ER- and PR-positive and HER2-negative, with negative sentinel lymph nodes bilaterally. In the absence of genomic recurrence testing, the decision to omit chemotherapy was based on the overall clinicopathological risk profile of both tumors. The patient underwent bilateral breast-conserving surgery followed by adjuvant radiotherapy and endocrine therapy. In the case reported by Peshin et al. (6), the bilateral tumors were also of the luminal subtype. Oncotype DX testing was performed to further assess recurrence risk and the potential benefit of adjuvant chemotherapy. Given the node-positive IDC and the overall clinicopathological risk profile, the multidisciplinary team also considered the patient eligible for adjuvant endocrine therapy combined with a CDK4/6 inhibitor based on evidence from the NATALEE trial. The case reported by Al-Bitar et al. (5) presented with bilateral luminal breast cancers with regional nodal involvement. Neoadjuvant chemotherapy was administered prior to definitive surgery, with the reported treatment considerations including tumor downstaging, optimization of the surgical outcomes, and assessment of chemosensitivity. Beyond nodal status, molecular subtype discordance may further influence the treatment approach. In the case reported by Rao et al. (4), the coexistence of luminal ILC and HER2-positive IDC, together with lymph node metastasis, was managed with chemotherapy combined with HER2-directed therapy. In the case reported by Copur et al. (8), node-positive triple-negative IDC coexisted with luminal ILC, and neoadjuvant chemotherapy was administered to achieve tumor downstaging and assess treatment response. In addition, Brock et al. (7) reported receptor discordance between the luminal primary tumor and its metastatic lymph nodes, which exhibited a triple-negative phenotype, further illustrating the complexity of systemic treatment selection. A case (10) involving bilateral HER2-positive tumors did not receive anti-HER2 targeted therapy, reflecting temporal factors related to the period of diagnosis, when HER2-directed agents, while available, had not yet achieved widespread adoption in adjuvant breast cancer practice. In contrast, another case presenting with bilateral HER2-positive tumors accompanied by extensive metastatic disease received palliative-intent chemotherapy combined with anti-HER2 targeted agents (9). Collectively, these cases underscore the importance of evaluating the clinicopathological characteristics of each tumor individually, particularly when histological or molecular discordance is present, with treatment strategies tailored according to comprehensive risk stratification incorporating tumor stage, grade, histology, molecular subtype, and nodal status of each tumor.

### Implications for follow-up

Among the eight sBPBC cases, two patients developed distant metastasis from the ILC components, one of which presented with bone metastasis 9 years after the initial diagnosis (4), while the other was found to have ILC metastasis to the cervix followed by metastasis to the bone, ovaries, and other distant sites (9). These observations are consistent with the recognized differences in the metastatic patterns between ILC and IDC. ILC is known to preferentially metastasize to the bones, gastrointestinal tract, peritoneum, retroperitoneum, and the reproductive system (particularly the ovaries) (21), whereas IDC typically metastasizes to the lungs and the liver (22). These differences in metastatic patterns highlight the importance of maintaining clinical vigilance during follow-up, particularly when patients develop new symptoms or signs potentially suggestive of recurrence or metastasis. Awareness of the distinct metastatic patterns of ILC and IDC may help clinicians select appropriate targeted diagnostic evaluations when relevant clinical concerns arise. A thorough understanding of the biological characteristics of each tumor component may facilitate appropriate clinical assessment and management.

### Limitations

Several limitations should be acknowledged. This is a single-case report, and only seven previously reported cases were identified, limiting the generalizability of the observations. Heterogeneity in the staging, treatment, and follow-up across reports, together with potential publication bias, further limits direct comparison among cases. In the present case, comparative genomic profiling and germline genetic testing were not performed, limiting assessment of tumor clonality and hereditary predisposition, respectively. In addition, the relatively short follow-up period of 12 months is insufficient to assess long-term oncological outcomes, particularly the possibility of late recurrence. Future larger-scale, multi-institutional studies incorporating comprehensive molecular and genetic characterization and long-term follow-up are warranted to better define the biological characteristics and optimal management of histologically discordant sBPBC.

## Conclusion

This case and the comparative synthesis of seven previously reported cases demonstrate substantial heterogeneity in the clinicopathological and molecular characteristics, treatment strategies, and outcomes of synchronous bilateral IDC and ILC. These findings support independent characterization of each breast lesion, individualized treatment planning based on the features of both tumors, and appropriate clinical vigilance during follow-up.

## Data Availability

The original contributions presented in the study are included in the article/supplementary material. Further inquiries can be directed to the corresponding author.

## References

[1] Schulze AK Hoskin TL Moldoveanu D Sturz JL Boughey JC . Tumor characteristics of bilateral breast cancer compared with unilateral breast cancer. Ann Surg Oncol. (2024) 31:947–56. doi: 10.1245/s10434-023-14451-x 37906382

[2] Tada K . Bilateral breast cancer. Am Surgeon™. (2025) 91:854–8. doi: 10.1177/00031348251323713 39982251

[3] The Society of Breast Cancer China Anti-Cancer Association Breast Oncology Group of the Oncology Branch of the Chinese Medical Association . Guidelines for breast cancer diagnosis and treatment by the China Anti-cancer Association (2026 edition). Chin Oncol. (2025) 35:1157–255. doi: 10.19401/j.cnki.1007-3639.2025.12.00

[4] Rao Y Zhan Q Li H . Bilateral primary breast cancer with discordance in molecular subtypes: a case report. Int J Breast Cancer. (2025) 2025:9082803. doi: 10.1155/ijbc/9082803 40589790 PMC12208771

[5] Al-Bitar A Nasra AB Alsaoub L Aldakak MA Saifo M . Synchronous bilateral breast cancer with heterogeneous histology: invasive ductal and lobular carcinoma – a case report. Case Rep Oncol. (2025) 18:1457–67. doi: 10.1159/000548545 41323054 PMC12659413

[6] Peshin S Iftekher R Iqbal R Singh S Nagaishwarya M . The optimal treatment of invasive ductal and lobular carcinoma occurring at the same time needs to be established. Eur J Case Rep Internal Med. (2025) 12:005074. doi: 10.12890/2025_005074 39926571 PMC11801504

[7] Brock CM Clippard L . When breast cancer gets complicated. A case report of synchronous bilateral breast cancers with discordant tumor markers from the primary to nodes with findings of a sentinel internal mammary subpectoral lymph node. J Surg Case Rep. (2019) 2019:rjz015. doi: 10.1093/jscr/rjz015 30788096 PMC6368207

[8] Copur MS Ramaekers R Gauchan D Crockett D Clark D . Synchronous bilateral breast cancer with discordant histology. Oncol (Williston Park). (2017) 31:274–277, 312 28412779

[9] Benkerroum Z Babahabib A Kouach J Chahdi H Al Bouzidi A Moussaoui Rehali D . Métrorragie révélatrice d’un cancer du sein bilatéral synchrone: à propos d’un cas. Gynécol Obstétrique Fertilité. (2014) 42:360–4. doi: 10.1016/j.gyobfe.2013.11.009 24411296

[10] Wang TS . Bilateral simultaneous primary breast cancer with different pathological types: a case report. J Hebei North Univ. (2006) 23(2):73. in Chinese. doi: 10.3969/j.issn.2095-1396.2006.02.034

[11] Corso G Shen S Criscitiello C Mukhtar R Gamble L Guerini Rocco E . Invasive lobular carcinoma: Strategies and perspectives from the lobular breast cancer research group. Cancer Treat Rev. (2025) 140:103001. doi: 10.1016/j.ctrv.2025.103001 40815983

[12] Reig B Heacock L . Invasive lobular carcinoma in the screening setting. J Breast Imaging. (2025) 7:3–15. doi: 10.1093/jbi/wbae082 39657621

[13] Corso G Shen S Criscitiello C Mukhtar R Gamble L Guerini Rocco E . Comprehensive molecular portraits of invasive lobular breast cancer. Cell. (2015) 163:506–19. doi: 10.1016/j.cell.2015.09.033 26451490 PMC4603750

[14] Yoon KH Ahn JH Kim JY Park HS Kim SI Park S . Impact of axillary burden on survival: a comparative study of invasive lobular carcinoma and invasive ductal carcinoma in early-stage breast cancer. Cancers. (2025) 17:1002. doi: 10.3390/cancers17061002 40149336 PMC11940394

[15] Sokolova A Johnstone KJ McCart Reed AE Simpson PT Lakhani SR . Hereditary breast cancer: syndromes, tumour pathology and molecular testing. Histopathology. (2023) 82:70–82. doi: 10.1111/his.14808 36468211 PMC10953374

[16] Bedrosian I Somerfield MR Achatz MI Boughey JC Curigliano G Friedman S . Germline testing in patients with breast cancer: ASCO–society of surgical oncology guideline. JCO. (2024) 42:584–604. doi: 10.1200/JCO.23.02225 38175972

[17] Alkner S Tang M-H Brueffer C Dahlgren M Chen Y Olsson E . Contralateral breast cancer can represent a metastatic spread of the first primary tumor: determination of clonal relationship between contralateral breast cancers using next-generation whole genome sequencing. Breast Cancer Res. (2015) 17:102. doi: 10.1186/s13058-015-0608-x 26242876 PMC4531539

[18] Han Z Yao L Fang Y Chen S Lian R Yao Y . Patient-derived organoid elucidates the identical clonal origin of bilateral breast cancer with diverse molecular subtypes. Front Oncol. (2024) 14:1361603. doi: 10.3389/fonc.2024.1361603 38800414 PMC11116675

[19] Imyanitov EN Hanson KP . Molecular pathogenesis of bilateral breast cancer. Cancer Lett. (2003) 191:1–7. doi: 10.1016/S0304-3835(02)00523-2 12609703

[20] Hamy AS Abécassis J Driouch K Darrigues L Vandenbogaert M Laurent C . Evolution of synchronous female bilateral breast cancers and response to treatment. Nat Med. (2023) 29:646–55. doi: 10.1038/s41591-023-02216-8 36879128 PMC10033420

[21] Yu B Yan L Wang H Yang J Yang J . Invasive lobular carcinoma of the breast: metastatic patterns and treatment modalities—a review. Front Oncol. (2025) 15:1631670. doi: 10.3389/fonc.2025.1631670 41079065 PMC12510823

[22] Nasrazadani A Li Y Tseng G Xavier J Lee AV Lucas PC . Metastatic behavior of mixed invasive ductal lobular carcinoma (Midc/ilc). JCO. (2020) 38:1085. doi: 10.1200/JCO.2020.38.15_suppl.1085

